# Assessment of epicardial adipose tissue thickness and the mean platelet volume in children with familial Mediterranean fever

**DOI:** 10.1186/s13052-015-0120-z

**Published:** 2015-02-22

**Authors:** Ünal Uluca, Fikri Demir, Aydın Ece, Velat Şen, Ali Güneş, Fesih Aktar, İlhan Tan, Duran Karabel, Ümitcan Yazgan, Muhammed Nurullah Sabaz

**Affiliations:** Department of Pediatrics, Dicle University Medical School, Diyarbakir, Turkey; Department of Pediatric Cardiology, Dicle University Medical School, Diyarbakir, Turkey; Department of Pediatric Rheumatology, Dicle University Medical School, Diyarbakir, Turkey; Department of Physiology, Dicle University Medical School, Diyarbakir, Turkey

**Keywords:** Familial Mediterranean fever, Epicardial adipose tissue thickness, Mean platelet volume, Atherosclerosis, Inflammation

## Abstract

**Background:**

Familial Mediterranean fever (FMF) is an inflammatory disease, which is suggested to be associated with increased risk of atherosclerosis. Epicardial adipose tissue (EAT) thickness and the mean platelet volume (MPV) are parameters used in prediction of atherosclerotic risk in various conditions. These parameters were evaluated in children with FMF and compared with healthy controls.

**Methods:**

Forty-five patients with FMF and 54 age- and gender-matched healthy controls were assessed. Duration of symptoms, age at diagnosis, duration of delay in diagnosis, frequency and duration of FMF attacks, disease severity scores, response to colchicine therapy, MEditerraneanFeVer (MEFV) gene mutations, and MPV values were recorded. EAT thicknesses were measured by echocardiography.

**Results:**

Epicardial adipose tissue thicknesses of the children with FMF were found to be significantly greater than that of controls (5.1 ± 1.4 vs. 4.5 ± 0.9 mm, p = 0.036). FMF patients had significantly higher MPV values compared with the controls (7.8 ± 1.1 vs. 7.3 ± 1.4 fl, p = 0.044). Age at diagnosis, duration of delay in diagnosis, and MPV values were found to be correlated with EAT thickness in the patient group (r = 0.49, p = 0.001 for the former parameters and r = 0.32, p = 0.04 for MPV).

**Conclusion:**

Epicardial adipose tissue thickness and MPV values seem to be increased in children with FMF. These findings may indicate an increased risk of atherosclerosis in FMF patients.

## Introduction

Familial Mediterranean fever (FMF) is the most common cause of hereditary recurrent fever. It is an auto-inflammatory disease, characterized by attacks of fever, abdominal pain, chest pain, arthritis, and skin rashes. Familial Mediterranean fever is common in people from Eastern Mediterranean region, especially in Sephardic Jewish, Armenian, Turkish, and Arabic population [1,2].

Systemic inflammation plays an important role in development and progression of atherosclerosis. The possibility of atherosclerosis was reported to be increased in cases with rheumatic diseases [1,3]. Case-control studies allowing evaluation of carotid intima media thickness and endothelial dysfunction found a predisposition of FMF patients to atherosclerosis [4,5]. Mean platelet volume (MPV) and epicardial adipose tissue (EAT) thickness are novel parameters used in prediction of the risk of atherosclerosis [3,6-8]. Albeit contradictory results have been reported, there are some studies investigating the usefulness of MPV in assessment of atherosclerotic risk in cases with FMF [6,9,10]. However, no study evaluating EAT thicknesses of FMF patients was reported so far.

In this study, we investigated whether EAT thickness and MPV values differ in cases with FMF, when compared with healthy controls and assessed the correlation between EAT and MPV.

## Methods

### Study population

Forty-five FMF patients diagnosed according to Tel-Hashomer criteria [11] and age- and gender-matched 54 healthy normal weighted controls were enrolled into the study. Patients who experienced an FMF attack within two weeks prior to admission, overweighed children, and cases with dyslipidemia or any coexistent inflammatory disease were excluded from the study. The study protocol was approved by the Dicle University Hospital Ethics Committee.

Data regarding age, gender, weight, height, body mass index (BMI) which was calculated as weight/(height)^2^ (kg/m^2^), and MPV values of the patients and controls were detected and recorded. Duration of symptoms, age at diagnosis, duration of delay in diagnosis, frequency and duration of FMF attacks, response to colchicine, and determined MEditerraneanFeVer (MEFV) gene mutations in FMF patients were recorded. The severity of disease was determined according to the criteria adapted by Ozen et al. [12] for affected children. Scores ranging from 3 to 5 points indicated mild disease, between 6 and 9 points showed moderate disease, and greater than 9 points was considered as severe disease.

### Measurement of epicardial adipose tissue thickness

All transthoracic echocardiographic examinations were performed by an experienced pediatric cardiologist (F.D.). A Vivid 5 Ultrasound System with appropriate transducers (GE Medical Systems, Horten, Norway) was used for echocardiographic evaluations. The epicardial adipose tissue was seen as an echo-free space between the outer surface of myocardium and visceral pericardium on two-dimensional echocardiography, and its thickness was measured on the free wall of right ventricle at end-diastole in parasternal long-axis view [13].

### Statistical analysis

The data were processed with SPSS (Statistical Package for Social Sciences) 16.0 program for Windows. Kolmogorov-Smirnov test was used to determine the distribution pattern of data. Normally distributed numerical variables were shown as mean ± standard deviation. Because of normal distribution, numeric variables of the patients and controls were compared with Student T-test. Kruskal-Wallis test was used for the comparison of three mutation-based patient subgroups. Chi-square test was used to compare categorical variables between the study groups. Correlations between numerical variables were evaluated by Pearson’s or Spearman’s correlation analysis. A p value less than 0.05 was accepted as statistically significant.

## Results

The study included 45 children with FMF (19 male, 26 female) and 54 healthy matched controls (24 male, 30 female). The main characteristics of the patient and the control groups are summarized in Table 1. The mean epicardial adipose tissue thicknesses of the patient and the control groups were 5.1 ± 1.4 and 4.5 ± 0.9 mm, respectively and the mean EAT thickness of the children with FMF were found significantly higher than that of controls (p = 0.036). FMF patients had significantly higher MPV values compared with the controls as well (7.8 ± 1.1 vs. 7.3 ± 1.4 fl, p = 0.044). There were no significant differences between the groups in terms of gender, age, or BMI (p > 0.05, for each comparisons). Age at diagnosis, duration of delay in diagnosis, and MPV values were found to be correlated with EAT thickness in the patient group (r = 0.49, p = 0.001 for the former parameters and r = 0.32, p = 0.04 for MPV). However, no correlation was found between disease severity score and EAT thickness. Forty-two of 45 FMF patients (93.3%) had a MEFV gene mutation. The patients were divided into three subgroups according to MEFV mutations (homozygote, heterozygote, and compound heterozygote) and the comparison of those subgroups in terms of EAT thickness and MPV revealed no significant difference (p > 0.05). Nine (21.4%) patients had M694V mutation and 33 (78.6%) patients with mutations other than M694V mutation such as V726A, M680I or R761H etc. Although, M694V mutation group had increased EAT (5.84 ± 1.94 vs. 4.79 ± 1.31 mm) and MPV (7.90 ± 0.96 vs. 7.73 ± 1.10 fl) values, while compared with the other MEFV mutations’ group, these differences did not reach to statistically significant levels (p = 0.107 and p = 0.497, respectively).

**Table 1:** **The main characteristics of the patients and the control group**

	**FMF group**	**Control group**	**p**
	**(n = 45)**	**(n = 54)**	
Gender (M/F)	19 / 26	24 / 30	NS
Age (years)	8.1 ± 4.1	7.9 ± 4.6	NS
BMI (kg/m^2^)	16.2 ± 2.3	16.3 ± 3.4	NS
EAT thickness (mm)	5.1 ± 1.4	4.5 ± 0.9	**0.036**
MPV (fl)	7.8 ± 1.1	7.3 ± 1.4	**0.044**
Onset of symptoms (years)	4.1 ± 3.0		
Age of diagnosis (years)	5.8 ± 3.0		
Delay in diagnosis (years)	1.7 ± 1.9		

BMI: Body mass index, EAT: Epicardial adipose tissue, FMF: Familial Mediterranean fever, F: Female, M: Male, MPV: Mean platelet volume, NS: Not significant.

## Discussion

Systemic inflammation is an important risk factor for atherosclerosis. Carotid intima media thickness, endothelial dysfunction, MPV and EAT thickness are some parameters that suggested for evaluation of increased atherosclerotic risk [3-5,7]. Familial Mediterranean fever is an inflammatory disease suggested to predispose to atherosclerosis [4-6]. The current study has found that FMF cases had significantly higher EAT thicknesses and MPV values when compared to healthy controls and EAT thickness has correlated with age of diagnosis, duration of delay in diagnosis, and MPV values.

Epicardial adipose tissue is a structure surrounding myocardium. It is composed mainly of adipocytes, but inflammatory, stromal, vascular, and nerve cells may also exist [14]. Epicardial adipose tissue expresses and secretes several bioactive molecules including interleukin (IL)-1, IL-1β, IL-6, IL-8, and IL-10, the levels of these interleukins were found as significantly higher in subjects with coronary artery disease when compared to healthy subjects [15]. Additionally, it was found that epicardial adipose tissue produces higher levels of inflammatory cytokines and reactive oxygen species than subcutaneous fat tissue in cases with coronary artery disease [16]. Recently, a meta-analysis of 16 studies revealed a correlation between EAT thickness and coronary artery disease [17]. Increased EAT volume was reported to be a stronger coronary risk factor than increased fat volume in any other part of the body. This may be related to proximity of EAT to coronary arteries. Possibly because of above-mentioned chronic low-grade inflammation and endothelial injury associated with reactive oxygen species, increased EAT seems to play important roles in pathogenesis of atherosclerosis.

Platelet activation has been suggested as a triggering factor in development of atherosclerosis and the mean platelet volume is accepted as a good indicator of platelet activation. CXCL4 is a chemokine of platelets having atherogenic activities by altering differentiation of T cells and macrophages, inhibiting apoptosis of neutrophils and monocytes, and stimulating CCL5. CCL5 is deposited on vascular endothelium and triggers atherogenic activities of monocytes. Platelet surface molecules such as GPIIb/IIIa, GP1bα, and P-selectin interact with endothelial cells, leukocytes and matrix components, which resultantly affect atherogenesis. Besides these, platelets also contribute to formation of lipid laden macrophages by modifying and endocytosing LDL particles [18,19].

Familial Mediterranean fever is an IL-1β dependent auto-inflammatory disease and is related to dysregulation of nod-like receptor family pyrin domain-containing 3 inflammasome (NLRP3) [20]. IL-6 and TNF-α levels were found higher in patients with FMF during both attack and attack-free period [21]. As mentioned above, inflammation is an important risk factor for atherosclerosis and EAT secretes various cytokines including IL-1β, IL-6, and TNF-α, the levels of which were significantly higher in patients with coronary artery disease [15]. The cause of increased EAT thickness in FMF cases remains unexplained. However, similarity in cytokine profiles of EAT and FMF disease makes us think that, these cytokines may have some roles in thickening of EAT and FMF. FMF may be associated with increased atherosclerotic risk through the cytokines released by thickened EAT and/or by inflammatory cells functioning in the pathogenesis of FMF. Platelet activation indicated by increased MPV values in our patients may also contribute to this atherosclerotic risk.

Mean platelet volume and EAT thickness are suggested to be used in prediction of the risk of atherosclerosis in various conditions including psoriatic arthritis, Behçet’s disease, ankylosing spondylitis, etc. [7,8,22]. Studies evaluating carotid intima media thickness and endothelial dysfunction in FMF patients have found an increased risk of atherosclerosis [4,5]. Usefulness of MPV in assessment of atherosclerotic risk in cases with FMF was also investigated. In accordance with our results, most of the studies found significantly higher MPV values in FMF patients and stated that FMF is associated with increased risk of atherosclerosis [6,9,10]. MPV values were found higher even in attack-free FMF cases. However, some other investigators did not determine any increase in platelet volumes [23,24]. Only one research reported lower MPV values in cases having FMF [25]. No study evaluated the atherosclerotic risk of FMF patients through measurement of EAT thickness so far. Atherosclerotic risk resulted from FMF-related chronic inflammation may be further increased by EAT thickness and MPV. We think the current investigation is unique because of simultaneous assessment of MPV values and EAT thicknesses in children with FMF. The finding that both of the parameters indicating atherosclerotic risk were increased in FMF patients may be associated with higher probability of atherogenesis. In previous studies, M694V mutation has been found together with severe disease and more frequent renal amyloidosis development in patients with FMF [2,12]. In our study, despite increased mean values of both EAT and MPV in M694V mutation group in comparison with other MEFV mutations’ group, these differences did not reach to significant levels, probably because of small number (n = 9) of patients in M694V subgroup. Furthermore, the mean age of our study group was 8.1 years; therefore these non-significant differences can reach to significant levels with increasing age especially for EAT. Further studies with more patients are needed to clarify this point.

Determination of increased risk of atherosclerosis in FMF patients even in childhood is important. MPV is a cheap and easily available parameter. Echocardiography provides simple and noninvasive assessment of EAT thickness. Given that children are expected to live long years; such an atherosclerotic risk may be associated with significant morbidity or mortality. Increased awareness about this risk may provide to take some measures for prevention of potential morbidities.

There are some limitations of the present study to be mentioned. The main limitation of the study is that parameters suggested to be related with atherosclerosis were evaluated cross-sectionally and follow-up measurements were not obtained. Relatively small sample size is the other limitation.

In conclusion, epicardial adipose tissue thickness and MPV values seem to be increased in children with FMF. We think EAT thickness and MPV are useful variables indicating the risk of atherosclerosis and may be evaluated in FMF patients of all ages. These findings may necessitate close follow-up and taking proper preventive measures.
